# Bacterial Cellulose Hybrid Composites with Calcium Phosphate for Bone Tissue Regeneration

**DOI:** 10.3390/ijms232416180

**Published:** 2022-12-19

**Authors:** Cristina Busuioc, Gabriela Isopencu, Adela Banciu, Daniel-Dumitru Banciu, Ovidiu Oprea, Alexandra Mocanu, Iuliana Deleanu, Mihaela Zăuleţ, Laura Popescu, Rodica Tănăsuică, Mihai Vasilescu, Anicuţa Stoica-Guzun

**Affiliations:** 1 Faculty of Chemical Engineering and Biotechnologies, University “Politehnica” of Bucharest, 1-7 Polizu Street, 011061 Bucharest, Romania; 2 Department of Biochemistry and Molecular Biology, University of Bucharest, 91-95 Splaiul Independentei, 050095 Bucharest, Romania; 3 Insitute of Hygiene and Public Veterinary Health, 5 Câmpul Mosilor Street, 021201 Bucharest, Romania; 4 Faculty of Physics, Babes-Bolyai University, 1 Mihail Kogalniceanu, 400084 Cluj-Napoca, Romania

**Keywords:** bacterial cellulose, 2,3-dialdehyde bacterial cellulose, calcium phosphate, hybrid composites

## Abstract

Bacterial cellulose (BC) is a unique microbial biopolymer with a huge number of significant applications in the biomedical field, including bone tissue engineering. The present study proposes to obtain and characterize BC hybrid composites with calcium phosphate as biocompatible and bioactive membranes for bone tissue engineering. BC precursor membranes were obtained in static culture fermentation, and after purification, were oxidized to obtain 2,3-dialdehyde bacterial cellulose (DABC). Calcium phosphate-BC oxidized membranes were produced by successive immersion in precursor solutions under ultrasonic irradiation. The samples were characterized for their physicochemical properties using scanning electron microscopy (SEM) coupled with energy-dispersive X-ray spectroscopy, attenuated total reflectance-Fourier transform infrared (ATR-FTIR) spectroscopy grazing incidence X-ray diffraction (GI-XRD), solid-state ^13^C nuclear magnetic resonance (CP/MAS ^13^C NMR), and complex thermal analysis. In vitro cell studies were also performed to evaluate the influence of modified morphological characteristics on cell adhesion and proliferation. The results showed an increase in porosity and biodegradability for DABC hybrid composites compared with BC. In vitro cell studies have revealed that both hybrid composites favor cell adhesion to the surface. The new BC and DABC hybrid composites with calcium phosphate could be considered promising materials for bone tissue regeneration.

## 1. Introduction

Bone is a complex structure that supports the bodies of vertebrates. To treat various bone diseases, among which fractures are the most common, auto/allografts or ceramic-coated/inert metallic implants are used because the bone’s ability to regenerate is limited [1,2]. In addition to the damage caused to those suffering from bone diseases, it should also be noted that the treatment of bone disease-related problems will create an estimated increase in medical costs of up to 25% by 2025 in the European Union [2]. 

Tissue engineering is an efficient alternative response to the challenges posed by bone regeneration methods. An ideal scaffold has not yet been discovered, but scientists have analyzed the most important features that a scaffold must fulfill. Biomaterials and their composites are good candidates that can mimic the three-dimensional architecture of an extracellular bone matrix. Different composite materials for bone tissue engineering have already been tested starting from synthetic and natural polymers, either used alone or in combination with inorganic compounds [3]. 

Among these materials, bacterial cellulose (BC) is considered a good candidate because it has some native properties that meet the requirements of many medical devices. For example, it is non-cytotoxic, biocompatible, and to some extent biodegradable, and it also can promote cellular interactions. BC could be considered a versatile material that can be adjusted by in situ or ex situ modification for specific tissue engineering applications [4,5]. Among these applications, bacterial nanocellulose is intensively studied for biocomposites used in hard tissue regeneration [6].

As a result of all these features, BC is already used in biomedicine, with some of its applications being wound dressing and artificial skin, vascular grafts and artificial blood vessel development, dental implants, bone and cartilage regeneration, peripheral nerve scaffolding, artificial cornea, and drug delivery, to list the most important of them [4,7].

It is important to mention that BC could be used for bone scaffolds in many forms including as native BC, dissolved in various solvents, as well as in the form of nanofibers and nanocrystals. For its application in bone tissue engineering, some drawbacks of BC must be eliminated. The first of them is its composition; BC is pure cellulose, and the human body is not able to degrade it because of the absence of cellulases. The biodegradability of BC, possible mechanisms for degradation, and possible solutions to enhance its biodegradability are largely discussed with the widening of the field of BC biomedical applications being of major interest nowadays [8]. 

In order to transform BC into a bioabsorbable material, methods have already been used to prove their efficiency such as oxidation using periodate to form biodegradable 2,3-dialdehyde bacterial cellulose (DABC), and TEMPO-mediated oxidation and cellulases incorporation in the BC matrix [1,9,10,11]. Using electron beam irradiation and γ-irradiation of BC membranes to control BC biodegradability for bone regeneration applications have also been tested and offer promising results [12,13].

The second problem concerning the structure of BC is its porosity; BC membranes have a dense structure with interconnected pores of small diameters (<100 nm), which prevents cell migration in the scaffold. To remove this disadvantage, in situ and ex situ modifications of BC were studied. 

For the in-situ modification of BC porosity, additional materials and substances were added to BC culture media, some examples being paraffin wax microsphere, gelatin microsphere, sand dollar skeleton, nanoparticles such as gold and hydroxyapatite (HA), and chondroitin sulfate [14,15,16,17,18,19]. The obtained scaffolds were tested for bone regeneration with promising results [16,17].

For the ex-situ modification of BC films, physical and chemical methods described in detail in many papers could be used [12,20,21,22,23]. For bone scaffolds, a very important aspect is the presence of bioactive components such as hydroxyapatite and tricalcium phosphate. 

Many chemical reactions have been performed to modify BC functionality and some of them have applications in bone tissue engineering. The reactions of oxidation using periodate and TEMPO-mediated oxidation have already been mentioned for BC biodegradability improvement. Phosphorylated BC samples have also been used to induce hydroxyapatite deposition to promote osteoblast proliferation and differentiation, the results showing that the nanocomposites BC–HA were more suitable for osteoblast adhesion [24]. 

BC was also used as a template to develop 3D-porous scaffolds for bone tissue engineering [25]. BC fibrils were used to obtain 3D-porous structures using a sol-gel technique [25]. For example, calcium phosphate was deposited on BC membranes under ultrasonic irradiation, followed by lyophilization and calcination in different conditions [26]. 

Among the great number of BC composites that have been tested as scaffolds for bone tissue engineering, those between BC and calcium phosphate stand out. In the large class of these mineral compounds, β-tri-calcium phosphate, octa-calcium phosphate, and hydroxyapatite could be given as examples that have been intensively tested for hard tissue engineering [27]. Since the inorganic part of the bone is mainly hydroxyapatite, many studies are dedicated to this material and especially to its composites in which some of its properties such as mechanical resistance, osteoconductivity, and osteoinductivity could be improved [28]. Among the natural polymers with which HA could be combined, BC has already passed experimental tests. The biocompatibility of the osteoblastic and stromal cells of bone marrow for BC–HA composites has also been tested with promising results [24,29,30,31,32]. Further, BC and oxidized BC membranes were mineralized with hydroxyapatite in order to obtain a more degradable and bioactive composite for bone regeneration [33]. 

This paper aimed to obtain BC and DABC composites with calcium phosphate in order to use these hybrid composites for bone tissue engineering. The materials were characterized with regard to their physicochemical properties and their in vitro activity. The novelty of this study consists of the comparison between BC and DABC hybrid composites in terms of biodegradability and biocompatibility. 

## 2. Results and Discussion

### 2.1. Characterization of BC–CP and DABC–CP Composites

Six samples were used in this study and their synthesis conditions and symbolic notations are depicted in Table 1. 

#### 2.1.1. Scanning Electron Microscopy

**Figure 1:**
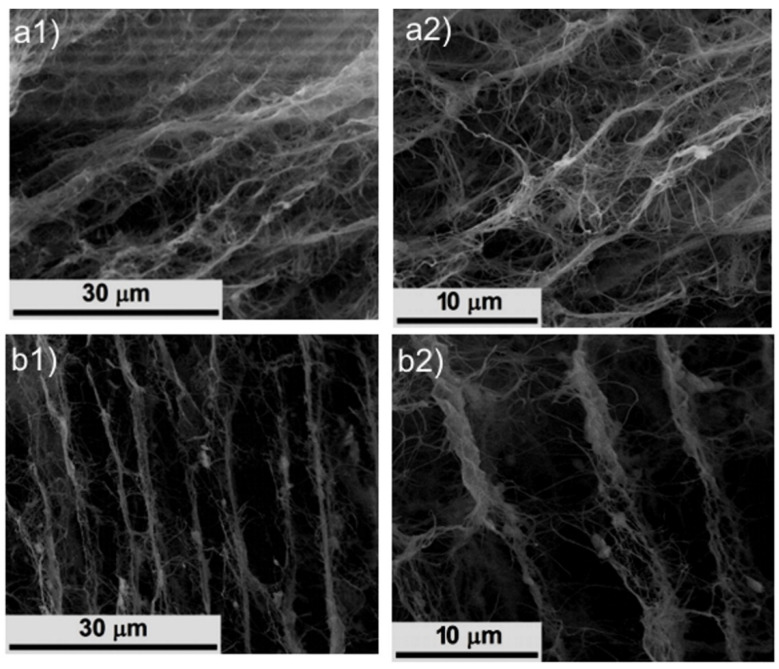
SEM images of native BC (**a1**,**a2**) and DABC (**b1**,**b2**) at different magnifications.

The SEM images presented in Figure 1 refer to cross-sections of native freeze-dried BC and DABC samples. The fibrillar structure with an entanglement of BC fibrils is visible in Figure 1(a1,a2) for native BC. DABC samples preserve the network structure of BC, but one can observe significant structural changes, especially in Figure 1(b2), where the fibrils are no longer intertwined, and so the pores are larger than those of the BC sample. This is one of the reasons why BC oxidation is used as a method to increase the porosity of BC. 

**Figure 2:**
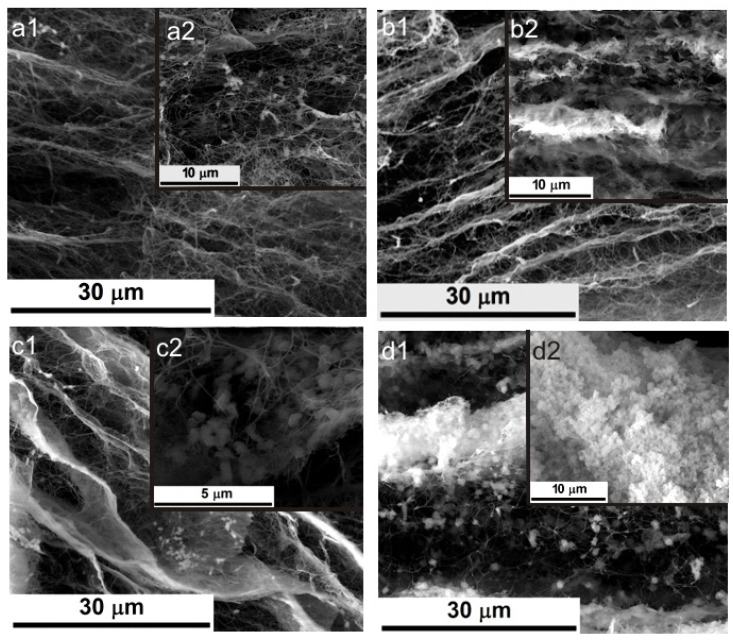
SEM images of native BC-PC1 (**a1**,**a2**), BC-PC2 (**b1**,**b2**), DABC-CP1 (**c1**,**c2**) and DABC-CP2 (**d1**,**d2**) at different magnifications.

Figure 2 presents SEM images of the BC and DABC samples after calcium phosphate deposition under US irradiation at two probe powers. The first conclusion is that the great power of the US horn favors CP deposition on BC and also on DABC. A higher density of CP particles is observed on DABC than on native BC. There are also differences between the form and the diameter of the particles deposited on BC and DABC. 

**Figure 3:**
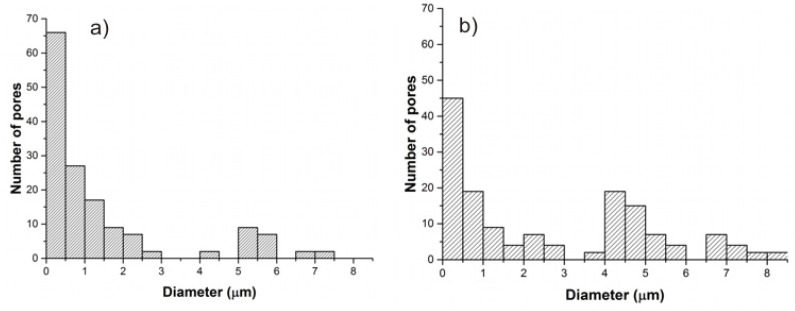
Pore size distribution of (**a**) BC, and (**b**) DABC samples.

SEM images were used to determine the pore size distribution of BC and DABC samples. To perform this analysis, ImageJ software (available at: https://imagej.nih.gov/ij/download.html (accessed on 20 November 2022) was used. For this analysis, the SEM images were converted to 8-bit binary images and these images were analyzed running <Analyze particles> in ImageJ. A detailed description of this procedure is given elsewhere [34,35]. The results obtained are depicted in Figure 3. 

In Figure 3, a change in pore size distribution between the two samples can be observed. BC has a large number of smaller pores in comparison to DABC. After oxidation, the number of small pores with a diameter of around 0.5 μm–1 μm decreases for DABC, and the number of pores with a diameter of around 4–6 μm increases. The changes in pore diameter distributions are a consequence of the oxidation process and confirm the SEM images presented in Figure 1. 

#### 2.1.2. FTIR Analysis and Solid-State ^13^C NMR Analysis

**Figure 4:**
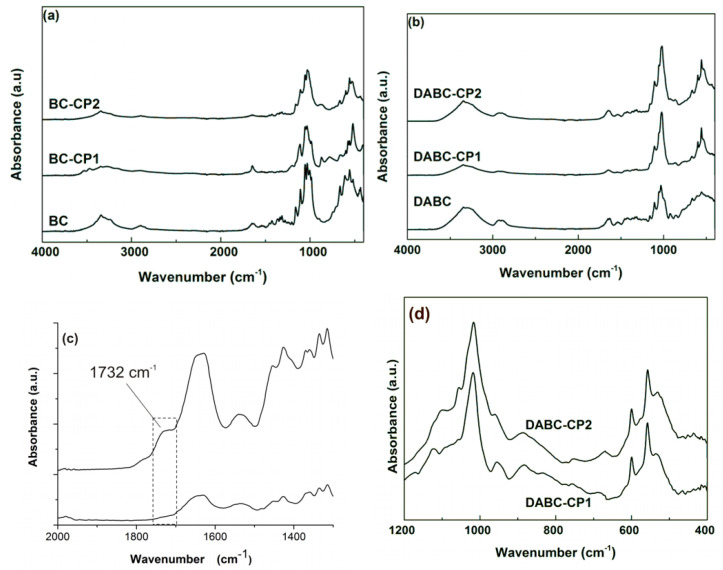
FTIR spectra of the studied samples: (**a**) BC, BC-CP1, and BC-CP2; (**b**) DABC, DABC-CP1, and DABC-CP2; (**c**) comparison between BC and DABC spectra in the range of 1400–2000 cm^−1^; and (**d**) subtracted spectra of DABC-CP1 and DABC-CP2 (reported to DABC).

The FTIR spectra of native BC and BC–calcium phosphate composites are presented in Figure 4a. The BC spectrum presents all of the characteristic vibrational modes of bacterial cellulose, some examples being 3342 cm^−1^ for O–H stretching vibrations, 2894 cm^−1^ for C–H stretching vibrations, and 1053 cm^−1^ for C–O–C pyranose ring skeletal vibrations. For oxidized BC (DABC), in the literature, there are two characteristic bands mentioned for aldehyde cellulose at 1740 cm^−1^ (carbonyl group stretch) and 880 cm^−1^ [11]. In our DABC spectrum, a new peak was visible at 860 cm^−1^, and another one at 1732 cm^−1^, this one is very weak but could be observed in Figure 3 in a comparison between the two spectra, one of BC and the other of DABC in the range of 1600–1800 cm^−1^. The intensity of this carbonyl band was also observed to remain quite low in other studies, even with prolonged oxidation times [36]. A decrease in the intensities of some absorption peaks at 1050 cm^−1^ and 1030 cm^−1^ was also observed in the FTIR spectrum of DABC. The phosphate ions PO_4_^3−^, which must be present in the composites with calcium phosphate, have characteristic bands in the range of 1200–550 cm^−1^. Some of them could be identified on the subtracted spectra of the samples containing calcium phosphate between (560–600 cm^−1^), 960 cm^−1^, and 1092 cm^−1^ (Figure 4d).

**Figure 5:**
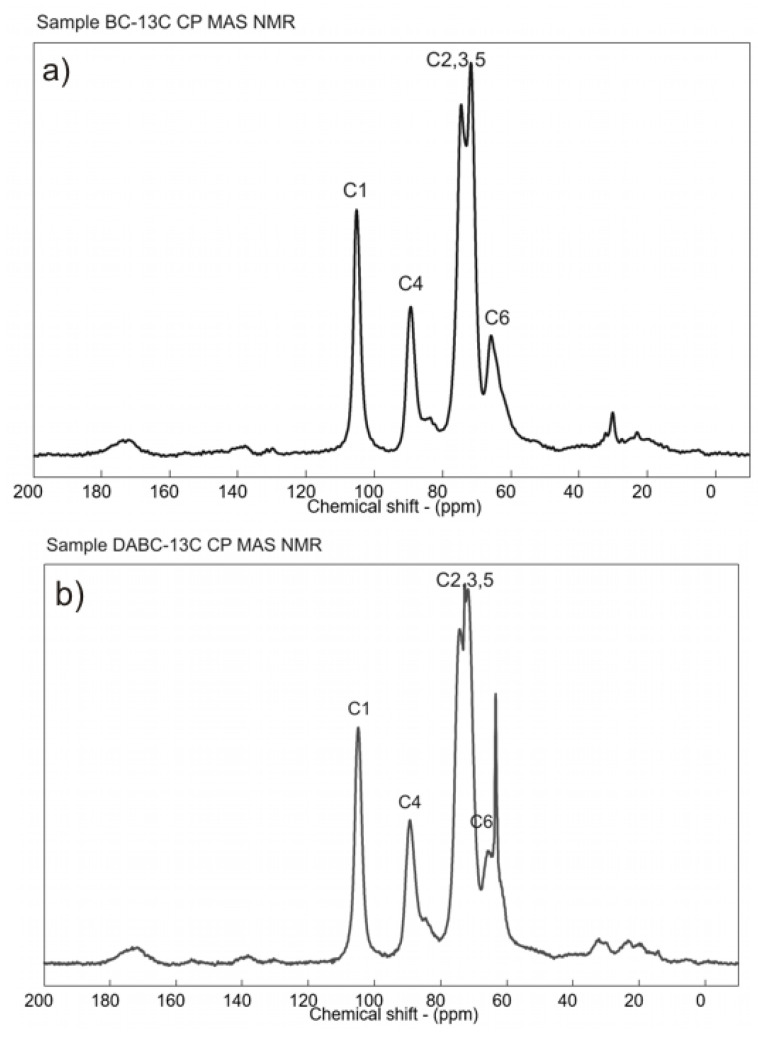
^13^C solid-state NMR spectra of (**a**) BC and (**b**) DABC.

The ^13^C CP MAS NMR spectra of BC and DABC are presented in Figure 5. The solid-state ^13^C-NMR spectrum of BC displays the characteristics signals of cellulose at 65.8 ppm associated with C6 carbon, between 71–75 ppm assigned to C2, C3, and C5 carbons, at 89.5 ppm associated with C4 carbon, and at 105 ppm assigned to C1 carbon [33,37]. In the solid-state ^13^C-NMR spectrum of DABC, the signals corresponding to the BC spectrum can be observed, but a series of small changes appear; first, all the signals have broader lines showing an increase in local disorder, and secondly, an increase in the signal at 85 ppm is observed. This signal is assigned to the C4 carbons of disordered cellulose chains indicating that the periodate oxidation reduced the BC crystallinity [36]. Other authors consider that resonances between 85–93 ppm could be assigned to aldehyde hydrates, i.e., the formation of hemialdal structures between the two newly formed aldehyde groups [38]. 

A resonance line at 63.66 ppm also appeared in the DABC spectrum. This region (between 60–70 ppm) is assigned to the hydroxymethyl C6 carbons and different authors consider that this signal represents the contribution of disordered chains, while the signal at 65 ppm arises from the crystalline core [36,37]. In addition to these, a resonance line can be observed at 72.95 ppm in the region assigned to C2, C3, and C5 carbons in the DABC spectrum. A possible explanation for this is that during the periodate oxidation of BC, the C2–C3 bonds in the glucose unit are broken, and the hydroxyl groups were converted to aldehyde groups [39]. 

#### 2.1.3. XRD

**Figure 6:**
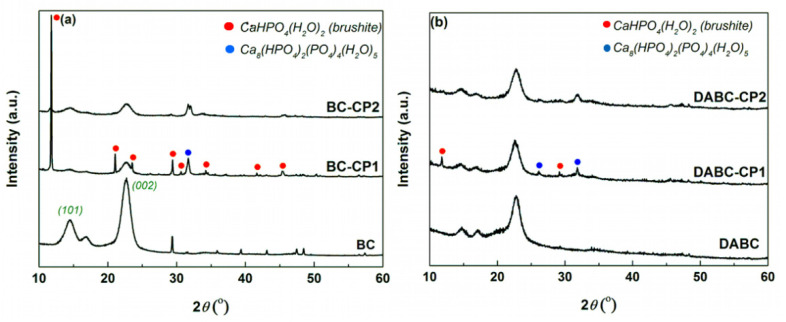
X-ray diffractograms of (**a**) BC, BC-CP1, and BC-CP2 and (**b**) DABC, DABC-CP1, and DABC-CP2.

The X-ray diffraction patterns of the studied samples are presented in Figure 6. The XRD spectrum of native BC presented in Figure 6a presents three strong Bragg peaks which were encountered at 14.4°, 16.9°, and 22.7°. These peaks indicated the presence of type I cellulose. The XRD pattern of the sample BC-CP1 presented some sharp peaks of brushite (calcium hydrogen phosphate hydrate, CaHPO_4_(H_2_O_2_)), monoclinic structure, (ICDD 00-072-0713), and a wider peak which was attributed to a complex phosphate (calcium hydrogen phosphate hydrate, Ca_8_(HPO_4_)_2_(PO_4_)_4_(H_2_O)_5_, (ICDD 00-079-0423); however, other low-crystallinity phosphates could be present in the composites, but they cannot be highlighted using this investigatory technique which is sensitive to long-range ordering. Referring to DABC-CP2, it seems that the number of mineral compounds decreases, probably as a consequence of ultrasound interference with the apatite nucleation processes. Such behavior can be explained on the grounds of pH influences during deposition, being well-known that small shifts in pH can lead to the crystallization of different calcium phosphates. 

The X-ray diffractograms of DABC, DABC-CP1 and DABC-CP2 are presented in Figure 6b. The first observation is that the characteristic peaks of BC are also present, but their intensity is decreased in the DABC pattern, indicating that the oxidation attacks the crystalline nucleus. In DABC-CP1 and DABC-CP2 XRD patterns, some peaks of the previously mentioned calcium phosphates could be observed, but with lower intensity as a sign of quantitative reduction, a fact which contradicts the SEM images to a certain extent but becomes explainable when the intrinsic inhomogeneous nature of BC is considered. 

#### 2.1.4. Thermogravimetric Analysis

**Figure 7:**
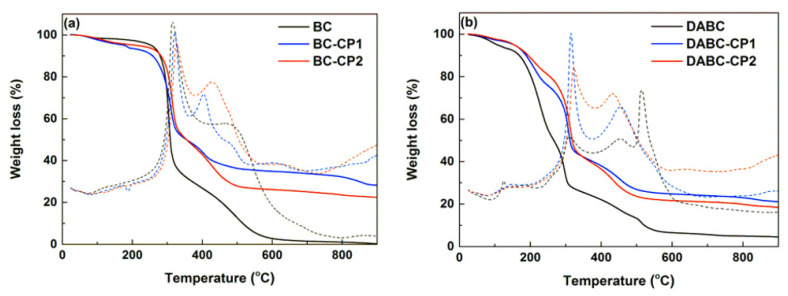
Thermogravimetric analysis (TG and DSC) of the studied samples: (**a**) BC, BC-CP1, and BC-CP2 and (**b**) DABC, DABC-CP1, and DABC-CP2.

The thermogravimetric curves for all studied samples are presented in Figure 7. From these, one can observe common features for all curves (TG and DSC). The first mass loss corresponds to the evaporation of light volatile compounds, especially water, a process accompanied by an endothermic effect on the DSC curve. A degradation process of the polymeric matrix (BC) takes place at approximately 135 °C, which is related to the depolymerization, dehydration, and decomposition of the glycosyl units, for which the mass loss is considerable, especially for the samples without calcium phosphates, the overall effect being an exothermic one. The last mass loss above 400 °C can be correlated with the formation of carbon residue in several stages, as confirmed by the exothermic effects. Based on thermogravimetric analysis, an estimation of the inorganic content (calcium phosphates) is given in Table 2. Thus, significant amounts of calcium phosphates deposed on BC and DABC can be observed. 

From the estimation of inorganic component content given in Table 2, one can see that on native BC, an increased amount of mineral phase was deposed compared to DABC, validating the data provided by the XRD patterns. The same phenomenon was reported by Hutchens et al. [40]. A possible explanation lies in the BC oxidation process to dialdehyde, which could affect apatite nucleation and restrict calcium and phosphate access during calcium phosphate precipitation. Moreover, US irradiation at 20% probe power ensures a better deposition yield that could reach an improvement of 25%.

### 2.2. Degradation of BC and DABC In Vitro

**Figure 8:**
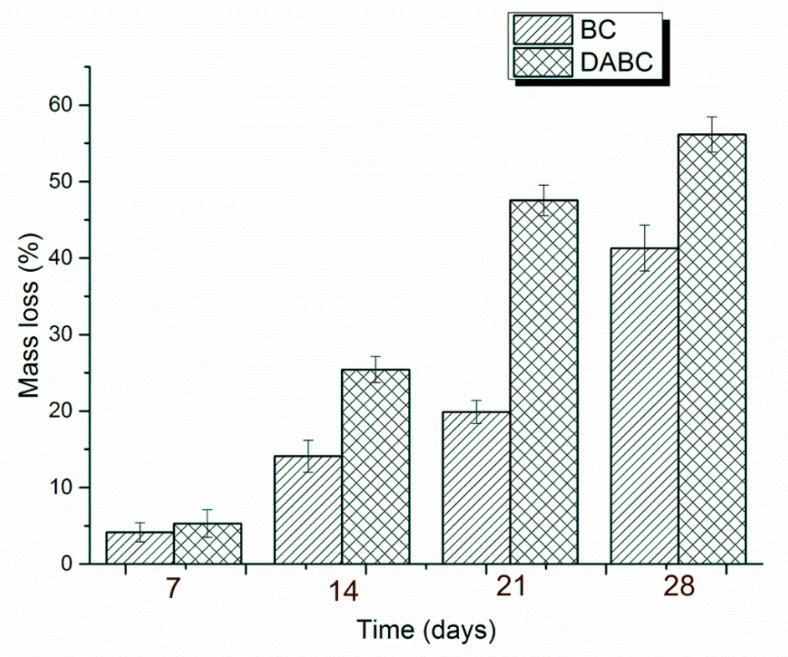
Percentage of mass loss for BC and DABC during in vitro degradation (n = 3; error bars = SD).

Data obtained from the in vitro degradation of BC and DABC are presented in Figure 8. After 28 days from the beginning of the experiment, it is obvious that the oxidized BC (DABC) lost more mass than the native BC. This fact is in accordance with other observations presented in the literature, the explanation being the breaking of the cellulose chains in the simulated physiological medium [41]. A DABC degradation mechanism was proposed by Hutchens et. al. (2009) [40]. The aforementioned authors considered that the main degradation products are 2,4-dihydroxybutyric acid, glycolic acid, and soluble carbohydrates. Huo et al. (2018) and Luz et al. (2020) confirmed that 2,4-dihydroxybutyric acid and glycolic acid are the main products of the hydrolytic degradation mechanism [33,42]. 

### 2.3. In Vitro Cell Studies

**Figure 9:**
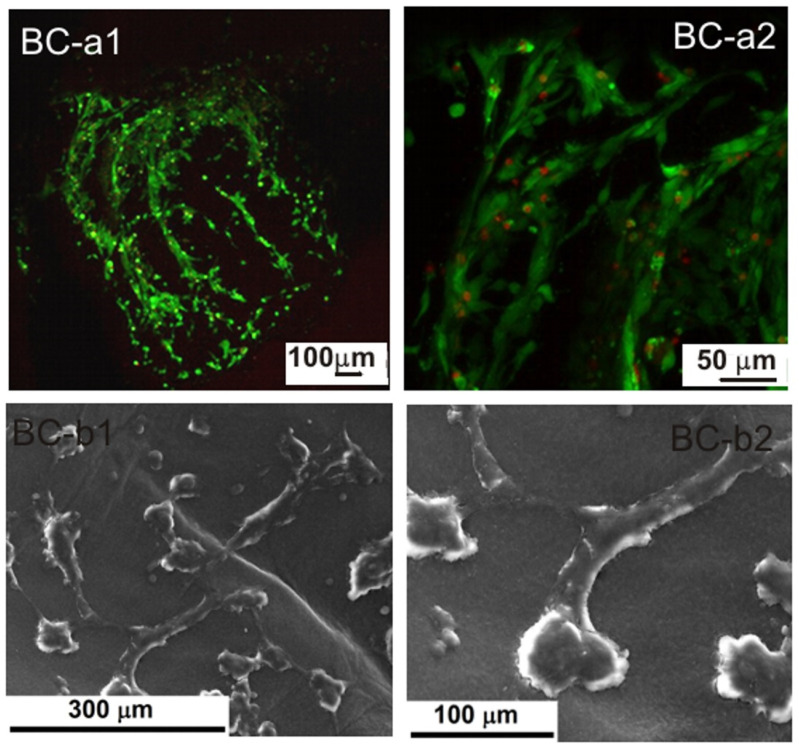
Fluorescence microscopy images (**a1**,**a2**) and SEM images (**b1**,**b2**) of the adherent cells on a BC sample.

**Figure 10:**
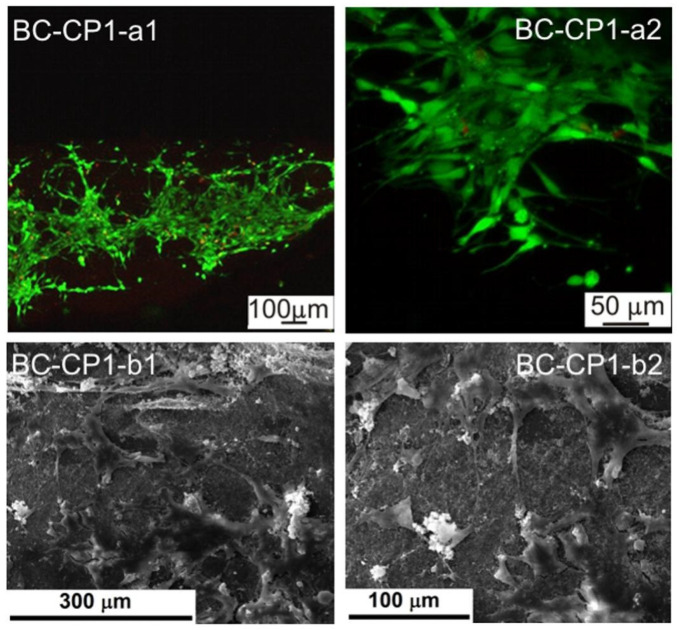
Fluorescence microscopy images (**a1**,**a2**) and SEM images (**b1**,**b2**) of the adherent cells on a BC-CP1 sample.

**Figure 11:**
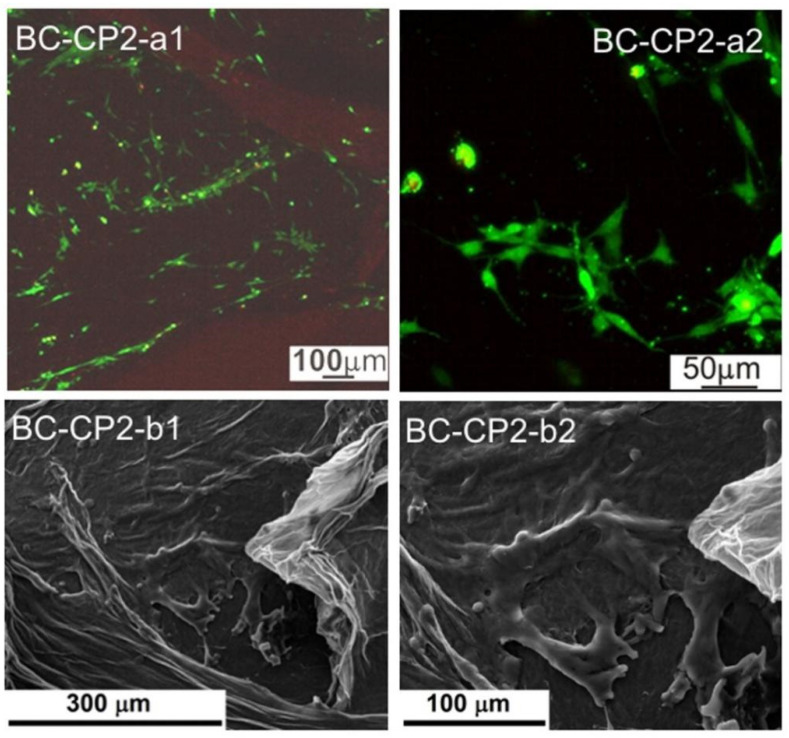
Fluorescence microscopy images (**a1**,**a2**) and SEM images (**b1**,**b2**) of the adherent cells in a BC-CP2 sample.

**Figure 12:**
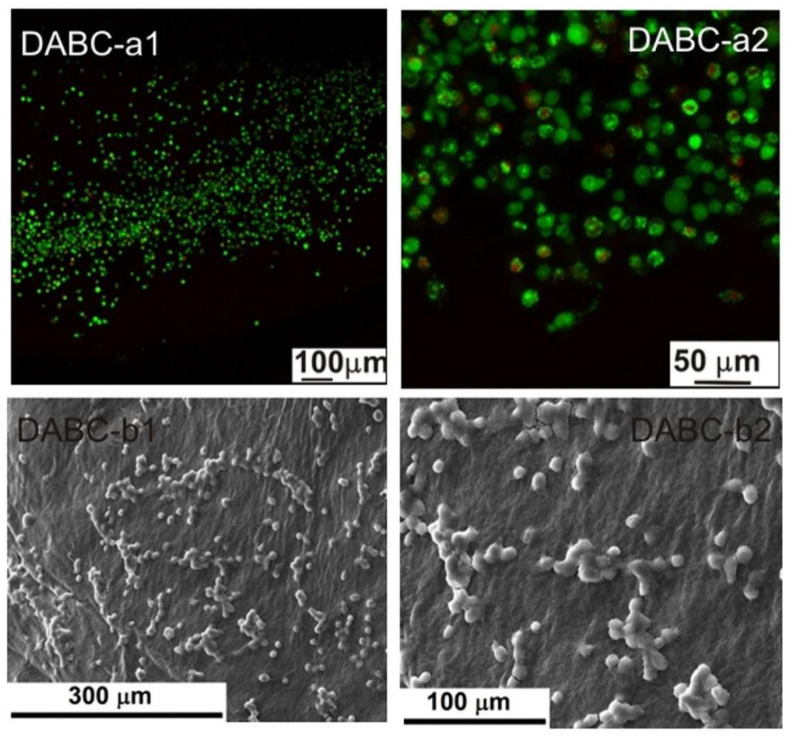
Fluorescence microscopy images (**a1**,**a2**) and SEM images (**b1**,**b2**) of the adherent cells in a DABC sample.

**Figure 13:**
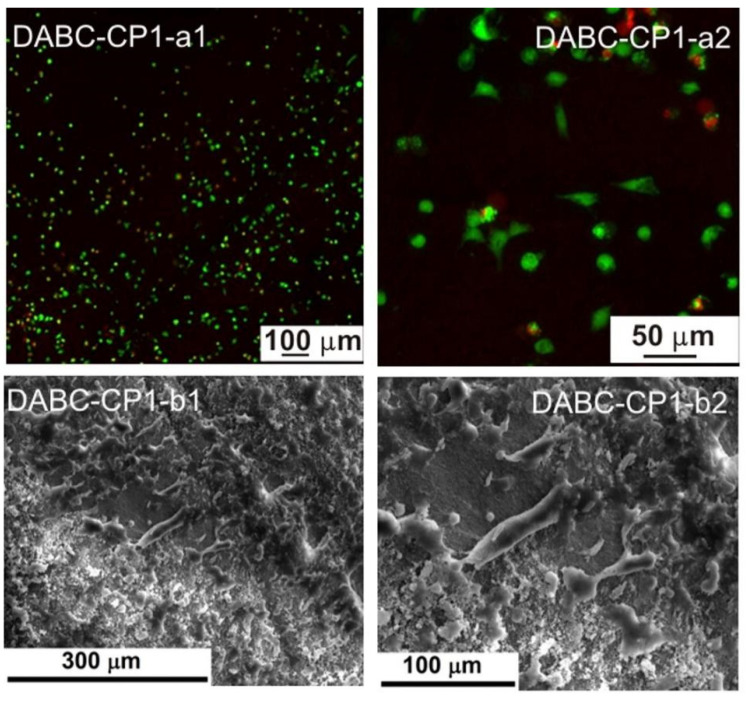
Fluorescence microscopy images (**a1**,**a2**) and SEM images (**b1**,**b2**) of the adherent cells in a DABC-CP1 sample.

**Figure 14:**
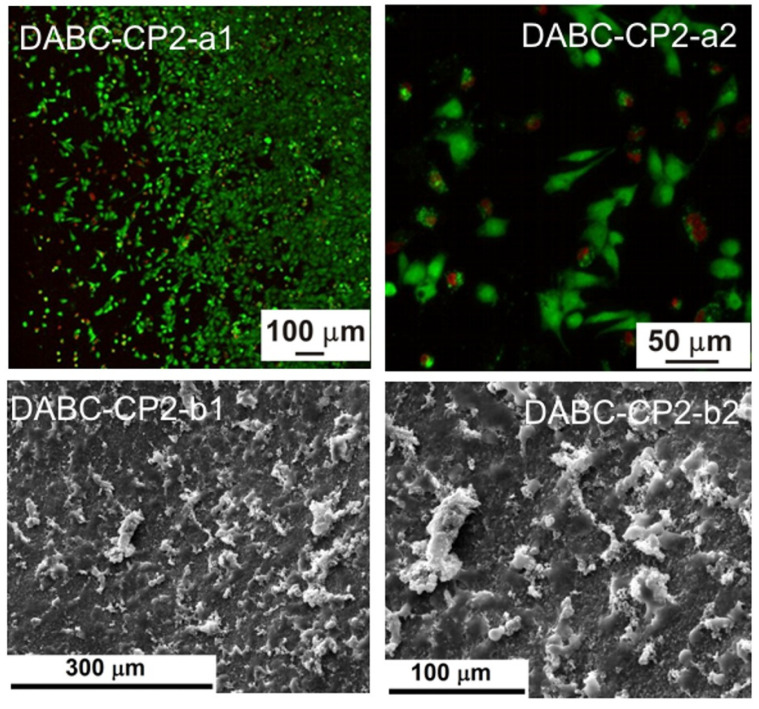
Fluorescence microscopy images (**a1**,**a2**) and SEM images (**b1**,**b2**) of the adherent cells in a DABC-CP2 sample.

The biological evaluation was performed through the cellular viability test coupled with fluorescence microscopy (FM), as well as by investigating the cell morphology with scanning electron microscopy (SEM). The resulting images are displayed in Figure 9, Figure 10, Figure 11, Figure 12, Figure 13 and Figure 14.

Based on the fluorescence microscopy images (Figure 9(a1,a2) and Figure 14(a1,a2)), the samples can be divided into two groups: (i) the ones containing BC (Figure 9(a1,a2) and Figure 11(a1,a2)), for which the cells seem to be more adherend to the surface, being grouped in large populations with a star-like shape, emitting extensions toward the neighboring cells, and creating extended networks in preferential directions; and (ii) the ones containing DABC (Figure 12(a1,a2) and Figure 14(a1,a2)), for which the cells have mainly a round shape and are lower in density, indicating that the culturing substrates did not ensure favorable conditions for adhesion and proliferation.

Basically, the adhesion to the substrate can be quantified according to the following criteria: (i) cell shape—rounded vs. flattened/angular/star-shaped; (ii) cell extensions—length and distribution; (iii) cell interpenetration—formation of extensive networks; and (iv) cell migration—preferential areas to which they move.

DABC and DABC-CP1 samples, in particular, exhibit a group of cells dispersedly located and round in shape. This fact could suggest that they did not adhere, or they adhered, divided, and are in the immediate state after division without migrating much. However, at longer stages, they could adopt a specific, flattened morphology, with extensions and cell–cell interactions.

The second series of images (Figure 9(a1,a2) and Figure 14(a1,a2)) were obtained after 24 h in contact with the cell culture, the cells being subsequently fixed with glutaraldehyde and the excess medium being extracted. Afterward, the samples were immersed in ethanol to shorten the drying process, tamped with absorbent paper, and dried in a vacuum; as a final step, the samples were coated with a thin layer of gold by DC magnetron sputtering to ensure surface conductivity. The presented images are compatible, to a certain extent, with the previously presented data, especially when it comes to shape. Thus, BC, BC-CP1, and BC-CP2 samples show large star-like cells frequently connected, DABC is covered with spherical cells gathered in small groups, while DABC-CP1 and DABC-CP2 are heavily and homogeneously populated by cells; indeed, their shape is not typical for a mature cell, instead, they appear flattened and seem to be in an incipient state of long-range organization. The explanation for such differences can be found in the investigating parameters, namely the surface area scanned by SEM compared to FM and the importance of position and depth in FM. In other words, it is strongly possible that the FM images were obtained from unfavorable areas where adhesion and proliferation did not occur.

In conclusion, acceptable cell viability was recorded for short stages, with the possibility for it to be improved over longer intervals. Otherwise, the cell morphology was sometimes specific to osteoblasts, creating intercellular networks based on the issued extensions.

Fluorescence microscopy was also employed for assessing the influence of the developed materials on the preosteoblasts at longer stages, namely 3 and 7 days (see Supplementary Material, Figures S1–S4). The results are quite similar to those displayed in Figure 9, Figure 10, Figure 11, Figure 12, Figure 13 and Figure 14: BC, BC-CP1, and BC-CP2 samples appear to ensure an adequate platform for cell proliferation, with an increase in cell population and intercellular connectivity from BC to BC-CP2, highlighting the beneficial effects of calcium phosphates deposition. Moreover, the cells maintain their spherical shape for the other three samples (DABC, DABC-CP1, and DABC-CP2), and the green color indicates the fact that they are alive, even after 7 days. It can also be noticed that the organization of cells into extended networks copies the microstructure of the substrate, a 3D scaffold with smaller or larger pores. Prolonging the contact time seems to favor the development of longer extensions between neighboring cells, which ensures rapid integration of the substitute material in the host body.

This study started from two major problems which are associated with the use of BC for bone scaffolds; the first is the lack of biodegradability in the human body and the second, is the porosity. As we have already pointed out, starting from the existing studies and our own research, BC has a network structure of interconnected pores with small diameters, and this could be a problem for cell migration in the scaffold. In this study, we chose the chemical modification of BC by periodate oxidation and to obtain a hybrid composite by depositing calcium phosphates using ultrasound irradiation. In order to highlight the properties of the new composites, samples with unmodified BC on which calcium phosphate was deposed were also studied. The first part of our study was dedicated to highlighting the existence of aldehyde groups in the DABC samples. FTIR and ^13^C CP MAS NMR spectra proved the chemical transformation of BC in DABC.

The comparison of the physicochemical properties of the two types of composites revealed significant differences. From the image analysis of SEM results, it was observed that DABC has a higher porosity than BC. This is an important fact because the exact purpose of BC oxidation is to increase porosity. From the thermogravimetric analysis, the inorganic content of the hybrid composites was estimated, and the conclusion was that a great amount of mineral phase was deposed on BC in comparison to DABC, even where the agglomeration of CPs was observed on DABC in the SEM images. US irradiation at lower power (20%) ensures a better CPs deposition yield on BC and DABC. 

Regarding in vitro degradation, DABC has a much higher degradation rate than BC. This confirms that BC oxidation using periodate to form 2,3-dialdehyde bacterial cellulose is a way to enhance its biodegradability, as per other studies in the literature [11,33]. The biological evaluation of the BC and DABC hybrid composite also reveals interesting results. BC composites seem to favor cell’s adhesion to the surface, and subsequently proliferation, slightly better than DABC composites, for which the cells are lower in density in the fluorescence microscopy images, but quite crowded in the SEM images, indicating reasonable cell viability at short stages, with the possibility of improvement over longer intervals.

If the results concerning biodegradation and biological evaluation are taken into account, the conclusion is that when the long-term use of the material is desired, BC hybrid composites are preferable. For short-term applications, DABC hybrid composites are preferable. However, both types of composites can be used in bone tissue engineering.

## 3. Materials and Methods

All reagents used were of analytical grade and all solutions were prepared using deionized water. Reagents were purchased from Sigma-Aldrich Chemie GmbH (Germany): calcium chloride (CAS-No-10043-52-4); sodium phosphate dibasic (CAS-No-7558-79-4); sodium (meta)periodate (CAS-No-7790-28-5); hydroxylamine hydrochloride (CAS-No-5470-11-1); sodium hydroxide (CAS-No-1310-73-2); RINGER tablets for the preparation of RINGER’S solution; and fructose (Cas-No-57-48-7).

### 3.1. Synthesis of Bacterial Cellulose 

The strain employed to produce cellulose was *Gluconacetobacter saccharivorans* isolated from apple vinegar. The selected cellulose-producing bacteria were examined by PCR followed by electrophoresis and sequencing for a specific fragment belonging to the 16S rRNA gene at the Department of Biochemistry and Molecular Biology (Faculty of Biology, Bucharest, Romania). 

The inoculum was prepared by growing *Gluconacetobacter saccharivorans* at 30 °C using a rotary shaker for 3 days. The inoculum was then transferred into a fermentation medium in 250 mL Erlenmeyer flasks in a ratio of 1:10. Fermentation was performed in a modified Hestrin–Schramm (MHS) medium containing 2% fructose as a carbon source. After 7 days at 27 °C, the pellicles formed in static culture were removed from the fermentation medium. Then, the obtained pellicles were treated with 0.5 N NaOH aqueous solution at 90 °C to remove bacterial cells from the BC membrane. The pellicles were then washed several times with distilled water until the water pH became neutral. For oxidation, the BC pellicles were used as wet membranes. 

### 3.2. Preparation of 2,3-Dialdehyde Bacterial Cellulose (DABC)

BC wet pellicles were immersed in an aqueous solution of sodium meta-periodate (1.3 g sodium meta-periodate added for 1 g BC dry) and gently stirred at 40 °C for 6 h in the dark. After this period, glycerol was used to decompose the sodium periodate. DABC membranes were washed with deionized water. The aldehyde content was determined using a Schiff base reaction with hydroxylamine [43] and the obtained value was 48 ± 0.24. 

### 3.3. Preparation of DABC–Calcium Phosphate Composites

Calcium phosphates (CPs) were deposited on the DABC membranes by alternating immersing cycles in calcium chloride solution (100 mM) and sodium phosphate dibasic solution (60 mM) under ultrasound irradiation using an Ultrasonic Processor VCX 500 (Sonics & Materials, Inc., USA), working at 500 W and 20 kHz. DABC membranes were immersed in calcium chloride solution for 20 min under US irradiation and then rinsed with deionized water and immersed in sodium phosphate dibasic solution for less than 20 min under US irradiation at 20 % and 40 % probe power. Pristine BC, as a reference sample, was used also for calcium phosphate deposition at the same conditions as the DABC samples. 

### 3.4. Characterization of BC–Calcium Phosphate Composites and DABC–Calcium Phosphate Composites

The final samples were characterized from a thermal, compositional, structural, morphological, and biological point of view. 

The morphology was investigated using scanning electron microscopy (SEM) with an FEI Quanta Inspect F50 microscope (Thermo Fisher Scientific, Waltham, MA, USA) equipped with an energy-dispersive X-ray spectroscopy (EDX) probe; the working distance was set at 10 mm and an accelerating voltage of 20 kV was used, the sample’s surface was coated with a thin layer of gold by DC magnetron sputtering for 40 s. 

The chemical bonds and groups were studied through attenuated total reflection-Fourier transform infrared (ATR-FTIR) spectroscopy with a Nicolet iS50 spectrophotometer (Thermo Fisher Scientific, Waltham, MA, USA), in the wavenumber range 400–4000 cm^−1^, 4 cm^−1^ resolution and 64 scans/sample. 

Solid-state CP/MAS ^13^C NMR measurements were performed at the Larmor frequency (150.92 MHz) using a Bruker Avance 600 spectrometer (14.10 T). The samples were packed in a ZrO rotor with an outer diameter of 3.2 mm. The CP/MAS spectra were recorded with a contact time of 2 ms. A chemical shift of the ^13^C nuclei was estimated by using an external reference of TMS (tetramethylsilane, δ = 0 ppm). A spinning rate of 10 KHz was used. 

The crystal structure and phase composition were studied by X-ray diffraction (XRD) with a PANalytical Empyrean diffractometer (Malvern Panalytical, Almelo, The Netherlands), using Ni-filtered Cu Kα radiation (λ = 1.54 Å), in the 2θ range of 10–60°, 2°/min scan speed, 0.02° step size, and 0.6 s preset time. 

The thermal analysis TG-DSC for the samples was performed with a Netzsch STA 449 C Jupiter apparatus. The samples were placed in an open crucible made of alumina and heated at 10 °C/min from room temperature to 900 °C under the flow of 50 mL/min dried air; an empty alumina crucible was used as a reference.

### 3.5. Degradation of BC and DABC In Vitro

Samples of BC and DABC cut in square shapes (10 × 10 mm) were incubated in physiological saline Ringier’s solution (pH = 7.4) at 37 °C for 28 days. The solution was renewed weekly. After 7, 14, 21, and 28 days, the samples were removed from the water, being dried, and weighed. The percentage of mass loss was determined according to the following equation:

(1) $$M\%=\frac{\left(M_{0}-M_{\tau }\right)\times 100}{M_{0}}$$

where *M*_0_ is the initial mass of the non-degraded sample, and *M_τ_* is the mass of the dried biodegraded sample after a specific period. All data were expressed as mean ± standard error mean.

### 3.6. In Vitro Cell Studies 

Preosteoblasts (MC3T3-E1 Subclone 4, ATCC CRL-2593) were cultured in MEM α (M8042, Sigma, St. Louis, MO, USA) supplemented with 10% Fetal Bovine Serum (FBS, F7524, Sigma, St. Louis, MO, USA), 1% L-glutamine and 1% Penicillin-Streptomycin (P4333, Sigma) at 37 °C, 5% CO_2_. The cells were seeded at a density of 3 × 10^5^ cells/cm^2^ on investigated materials, left to adhere for 20 min, and maintained for 24 h in the culture medium. 

The cellular viability was evaluated with calcein-AM (LIVE/DEAD Viability/Cytotoxicity Kit for mammalian cells, L3224, Life Technologies, Waltham, MA, USA) and propidium iodide. After 24 h in culture, the samples were washed with 1× Phosphate Buffered Saline (PBS, P3813, Sigma), and incubated for 40 min at room temperature with 1 µM calcein-AM and 1 µg/mL propidium iodide. Viability was assessed by fluorescence microscopy with a Zeiss LSM 880 confocal system (Zeiss, Oberkochen, Germany) with 488 and 514 nm lasers, and images were processed with ZEN 2.3 software (Zeiss, Oberkochen, Germany).

## 4. Conclusions

In this study, comparative data on hybrid composites with calcium phosphate of BC and DABC were presented. The conclusion is that by oxidizing BC to form 2,3-dialdehyde bacterial cellulose, several of its properties can be improved. In vivo studies are also needed to evidence the best applications for bone tissue regeneration of these composites, BC and DABC. 

## Figures and Tables

**Table 1:** Synthesis conditions for all the samples and their symbolic notations.

Sample Abbreviation	Description of the Sample
BC	Native BC purified
DABC	2,3-dialdehyde bacterial cellulose
BC-CP1	Calcium phosphate deposed on BC under US irradiation at 20% probe powder
BC-CP2	Calcium phosphate deposed on BC under US irradiation at 40% probe powder
DABC-CP1	Calcium phosphate deposed on DABC under US irradiation at 20% probe powder
DABC-CP2	Calcium phosphate deposed on DABC under US irradiation at 20% probe powder

**Table 2:** Centralized data from thermogravimetric curves of the studied samples.

Sample	Residual Mass (%)	Estimation of CP Mass (%)
BC	0.30 ± 0.01	-
BC-CP1	28.25 ± 1.50	28.03 ± 2.25
BC-CP2	22.49 ± 2.00	22.26 ± 1.78
DABC	4.57 ± 0.22	-
DABC-CP1	21.15 ± 1.76	17.37 ± 1.04
DABC-CP2	18.49 ± 0.92	14.59 ± 1.20

## Data Availability

Not applicable.

## Supplementary Materials

The following supporting information can be downloaded at: https://www.mdpi.com/article/10.3390/ijms232416180/s1.

## References

[1] Torgbo S., Sukyai P. (2018). Bacterial cellulose-based scaffold materials for bone tissue engineering. Appl. Mater. Today.

[2] Basu P., Saha N., Alexandrova R., Saha P. (2019). Calcium Phosphate Incorporated Bacterial Cellulose-Polyvinylpyrrolidone Based Hydrogel Scaffold: Structural Property and Cell Viability Study for Bone Regeneration Application. Polymers.

[3] Basu P., Saha N., Saha P. (2019). Swelling and rheological study of calcium phosphate filled bacterial cellulose-based hydrogel scaffold. J. Appl. Polym. Sci..

[4] Carvalho T., Guedes G., Sousa F.L., Freire C.S.R., Santos H.A. (2019). Latest Advances on Bacterial Cellulose-Based Materials for Wound Healing, Delivery Systems, and Tissue Engineering. Biotechnol. J..

[5] Torres F.G., Commeaux S., Troncoso O.P. (2012). Biocompatibility of Bacterial Cellulose Based Biomaterials. J. Funct. Biomater..

[6] Kumar A., Han S.-S. (2021). Efficacy of Bacterial Nanocellulose in Hard Tissue Regeneration: A Review. Materials.

[7] Wahid F., Huang L.-H., Zhao X.-Q., Li W.-C., Wang Y.-Y., Jia S.-R., Zhong C. (2021). Bacterial cellulose and its potential for biomedical applications. Biotechnol. Adv..

[8] Torgbo S., Sukyai P. (2020). Biodegradation and thermal stability of bacterial cellulose as biomaterial: The relevance in biomedical applications. Polym. Degrad. Stabil..

[9] Luo H., Xiong G., Hu D., Ren K., Yao F., Zhu Y., Gao C., Wan Y. (2013). Characterization of TEMPO-oxidized bacterial cellulose scaffolds for tissue engineering applications. Mater. Chem. Phys..

[10] Hu Y., Catchmark J.M. (2011). In vitro biodegradability and mechanical properties of bioabsorbable bacterial cellulose incorporating cellulases. Acta Biomater..

[11] Li J., Wan Y., Li L., Liang H., Wang J. (2009). Preparation and characterization of 2,3- dialdehyde bacterial cellulose for potential biodegradable tissue engineering scaffolds. Mater. Sci. Eng. C.

[12] An S.-J., Lee S.-H., Huh J.-B., Jeong S.I., Park J.-S., Gwon H.-J., Kang E.-S., Jeong C.-M., Lim Y.-M. (2017). Preparation and Characterization of Resorbable Bacterial Cellulose Membranes Treated by Electron Beam Irradiation for Guided Bone Regeneration. Int. J. Mol. Sci..

[13] Czaja W., Kyryliouk D., DePaula C.A., Buechter D.D. (2014). Oxidation of γ-irradiated microbial cellulose results in bioresorbable, highly conformable biomaterial. J. Appl. Polym. Sci..

[14] Stumpf T.R., Yang X., Zhang J., Cao X. (2018). In situ and ex situ modifications of bacterial cellulose for applications in tissue engineering. Mat. Sci. Eng. C.

[15] Barreiro A.M., Recouvreux D.O.S., Hotza D., Porto L.M., Rambo C.R. (2010). Sand dollar skeleton as templates for bacterial cellulose coating and apatite precipitation. J. Mater. Sci..

[16] Huang C., Ye Q., Dong J., Li L., Wang M., Zhang Y., Zhang Y., Wang X., Wang P., Jiang Q. (2023). Biofabrication of natural Au/bacterial cellulose hydrogel for bone tissue regeneration via in-situ fermentation. Smart Mater. Med..

[17] Niamsap T., Lam N.T., Sukyai P. (2019). Production of hydroxyapatite-bacterial nanocellulose scaffold with assist of cellulose nanocrystals. Carbohydr. Polym..

[18] Bayir E., Bilgi E., Hames E.E., Sendemir A. (2019). Production of hydroxyapatite–bacterial cellulose composite scaffolds with enhanced pore diameters for bone tissue engineering applications. Cellulose.

[19] De Olyveira G.M., Basmaji P., Costa L.M.M., dos Santos M.L., dos Santos Riccardi C., Guastaldi F.P.S., Scarel-Caminaga R.M., de Oliveira Capote T.S., Pizoni E., Guastaldi A.C. (2017). Surface physical chemistry properties in coated bacterial cellulose membranes with calcium phosphate. Mat. Sci. Eng. C.

[20] Xiong G., Luo H., Zhu Y., Raman S., Wan Y. (2014). Creation of macropores in three-dimensional bacterial cellulose scaffold for potential cancer cell culture. Carbohydr. Polym..

[21] Ahn S.-J., Shin Y.M., Kim S.E., Jeong S.I., Jeong J.-O., Park J.-S., Gwon H.-J., Seo D.E., Nho Y.-C., Kang S.S. (2015). Characterization of Hydroxyapatite-coated Bacterial Cellulose Scaffold for Bone Tissue Engineering. Biotechnol. Bioprocess Eng..

[22] Gorgieva S., Trček J. (2019). Bacterial Cellulose: Production, Modification and Perspectives in Biomedical Applications. Nanomaterials.

[23] Aditya T., Allain J.P., Jaramillo C., Restrepo A.M. (2022). Surface Modification of Bacterial Cellulose for Biomedical Applications. Int. J. Mol. Sci..

[24] Fang B., Wan Y.Z., Tang T.T., Gao C., Dai K.R. (2009). Proliferation and osteoblastic differentiation of human bone marrow stromal cells on hydroxyapatite/bacterial cellulose nanocomposite scaffolds. Tissue Eng..

[25] Luo H., Ji D., Li W., Xiao J., Li C., Xiong G., Zhu Y., Wan Y. (2016). Constructing a highly bioactive 3D nanofibrous bioglass scaffold via bacterial cellulose-templated sol-gel approach. Mater. Chem. Phys..

[26] Busuioc C., Ghitulica C., Stoica A., Stroescu M., Voicu G., Ionita V., Averous L., Jinga S. (2018). Calcium phosphates grown on bacterial cellulose template. Ceram. Int..

[27] Denry I., Kuhn L.T. (2016). Design and characterization of calcium phosphate ceramic scaffolds for bone tissue engineering. Dental Mater..

[28] Ielo I., Calabrese G., De Luca G., Conoci S. (2022). Recent Advances in Hydroxyapatite-Based Biocomposites for Bone Tissue Regeneration in Orthopedics. Int. J. Mol. Sci..

[29] Saska S., Barud H.S., Gaspar A.M.M., Marchetto R., Ribeiro S.J.L., Messaddeq Y. (2011). Bacterial Cellulose-Hydroxyapatite Nanocomposites for Bone Regeneration. Int. J. Biomat..

[30] Grande C.J., Torres F.G., Gomez C.M., Bañó M.C. (2009). Nanocomposites of bacterial cellulose/hydroxyapatite for biomedical applications. Acta Biomater..

[31] Zimmermann K.A., LeBlanc J.M., Sheets K.T., Fox R.W., Gatenholm P. (2011). Biomimetic design of a bacterial cellulose/ hydroxyapatite nanocomposite for bone healing applications. Mat. Sci. Eng. C.

[32] Luz E.P.C.G., Borges M.F., Andrade F.K., de Freitas Rosa M., Infantes-Molina A., Rodríguez-Castellón E., Vieira R.S. (2018). Strontium delivery systems based on bacterial cellulose and hydroxyapatite for guided bone regeneration. Cellulose.

[33] Luz E.P.C.G., Chaves P.H.S., Vieira L.A.P., Ribeiro S.F., Borges M.F., Andrade F.K., Muniz C.R., Infantes-Molina A., Rodríguez-Castellón E., de Freitas Rosa M. (2020). In vitro degradability and bioactivity of oxidized bacterial cellulose hydroxyapatite Composites. Carbohydr. Polym..

[34] Haeri M., Haeri M. (2015). ImageJ Plugin for Analysis of Porous Scaffolds used in Tissue Engineering. J. Open Res. Softw..

[35] AlMarzooqi F.A., Bilad M.R., Mansoor B., Arafat H.A. (2016). A comparative study of image analysis and porometry techniques for characterization of porous membranes. J. Mater. Sci..

[36] Azzam F., Galliot M., Putaux J.-L., Heux L., Jean B. (2015). Surface peeling of cellulose nanocrystals resulting from periodate oxidation and reductive amination with water-soluble polymers. Cellulose.

[37] Zhang L., Yan P., Li Y., He X., Dai Y., Tan Z. (2020). Preparation and antibacterial activity of a cellulose-based Schiff base derived from dialdehyde cellulose and L-lysine. Ind. Crops Prod..

[38] Guigo N., Mazeau K., Putaux J.-L., Heux L. (2014). Surface modification of cellulose microfibrils by periodate oxidation and subsequent reductive amination with benzylamine: A topochemical study. Cellulose.

[39] Wang I., Xiao G., Peng Y., Chen L., Fu S. (2019). Effects of cellulose nanofibrils on dyaldehyde carboxymethyl cellulose based dual responsive self-healing hydrogel. Cellulose.

[40] Hutchens S.A., Benson R.S., Evans B.R., Rawn C.J., O’Neill H. (2009). A resorbable calcium-deficient hydroxyapatite hydrogel composite for osseous regeneration. Cellulose.

[41] Favi P.M., Ospina S.P., Kachole M., Gao M., Atehortua L., Webster T.J. (2016). Preparation and characterization of biodegradable nano hydroxyapatite–bacterial cellulose composites with well-defined honeycomb pore arrays for bone tissue engineering applications. Cellulose.

[42] Hou Y., Wang X., Yang J., Zhu R., Zhang Z., Li Y. (2018). Development and biocompatibility evaluation of biodegradable bacterial cellulose as a novel peripheral nerve scaffold. J. Biomed. Mater. Res..

[43] Wu J., Zheng Y., Yang Z., Lin Q., Qiao K., Chen X., Peng Y. (2014). Influence of dialdehyde bacterial cellulose with the nonlinear elasticity and topology structure of ECM on cell adhesion and proliferation. RSC Adv..

