# Supplementary material for: Bacterial Cellulose Hybrid Composites with Calcium Phosphate for Bone Tissue Regeneration
Source: Int J Mol Sci. 2022 Dec 19;23(24):16180. doi: 10.3390/ijms232416180 (PMC9784094; doi:10.3390/ijms232416180)
Supplement: Supplementary file 1 [file ijms-23-16180-s001.zip › ijms-2034846-supplementary.pdf]

Supplementary Material- Bacterial cellulose hybrid composites with calcium phosphate for bone tissue regeneration

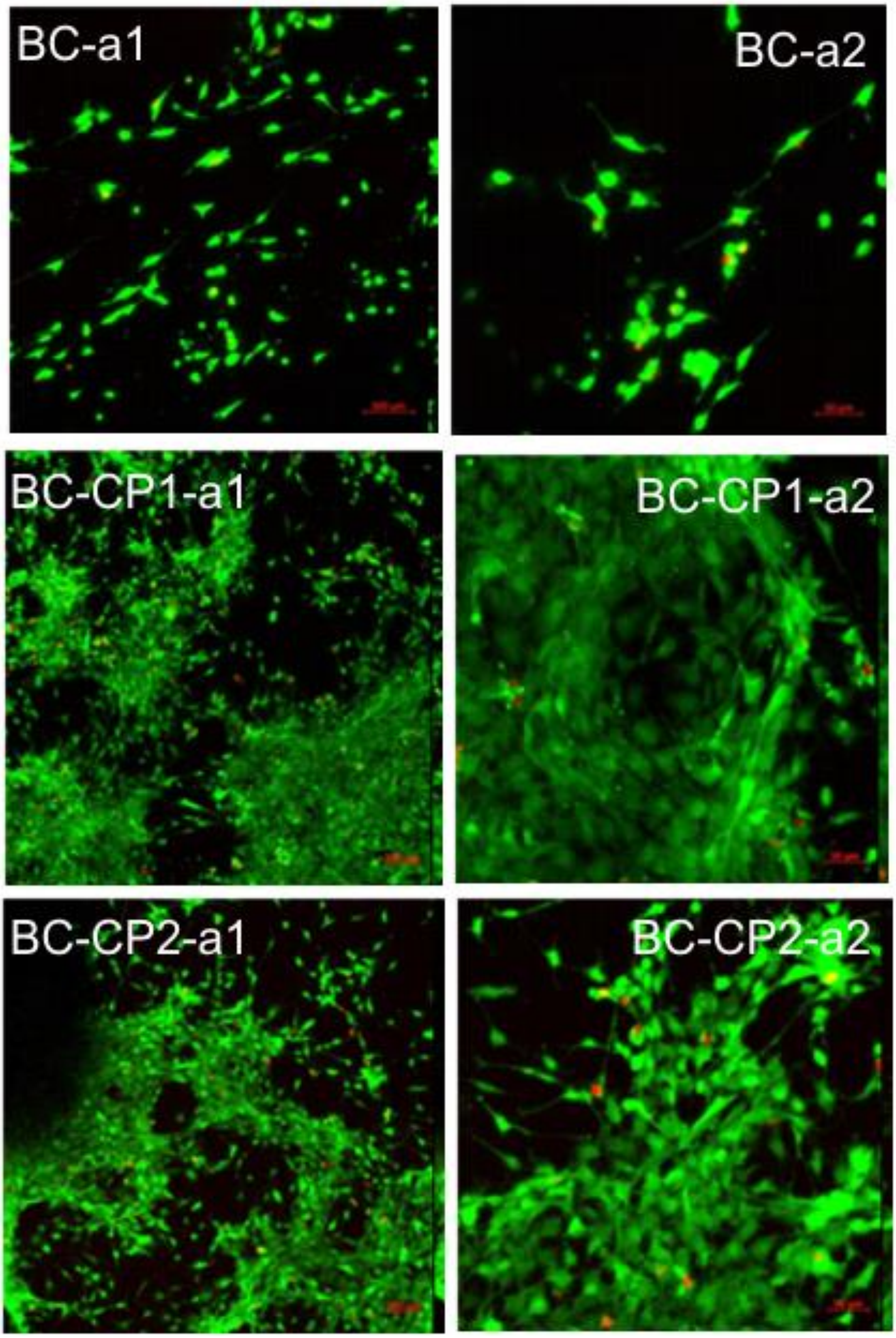

Figure S1. Fluorescence microscopy images at different magnifications: a1 (100μm) and a2 (50μm) of the adherent cells on BC, BC-CP1 and BC-CP2 samples after 3 days.

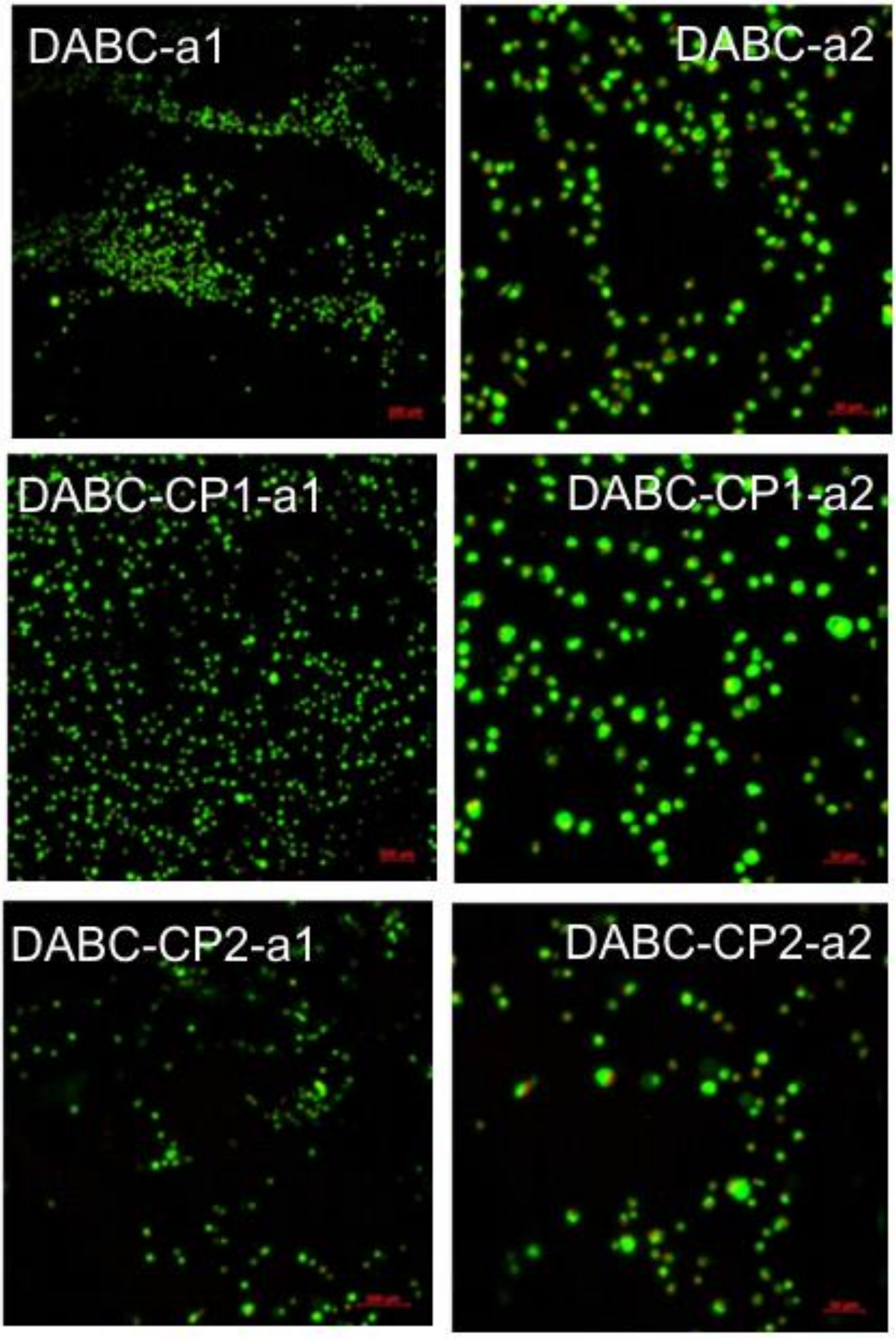

Figure S2. Fluorescence microscopy images at different magnifications: a1 (100μm) and a2 (50μm) of the adherent cells on DABC, DABC-CP1 and DABC-CP2 samples after 3 days.

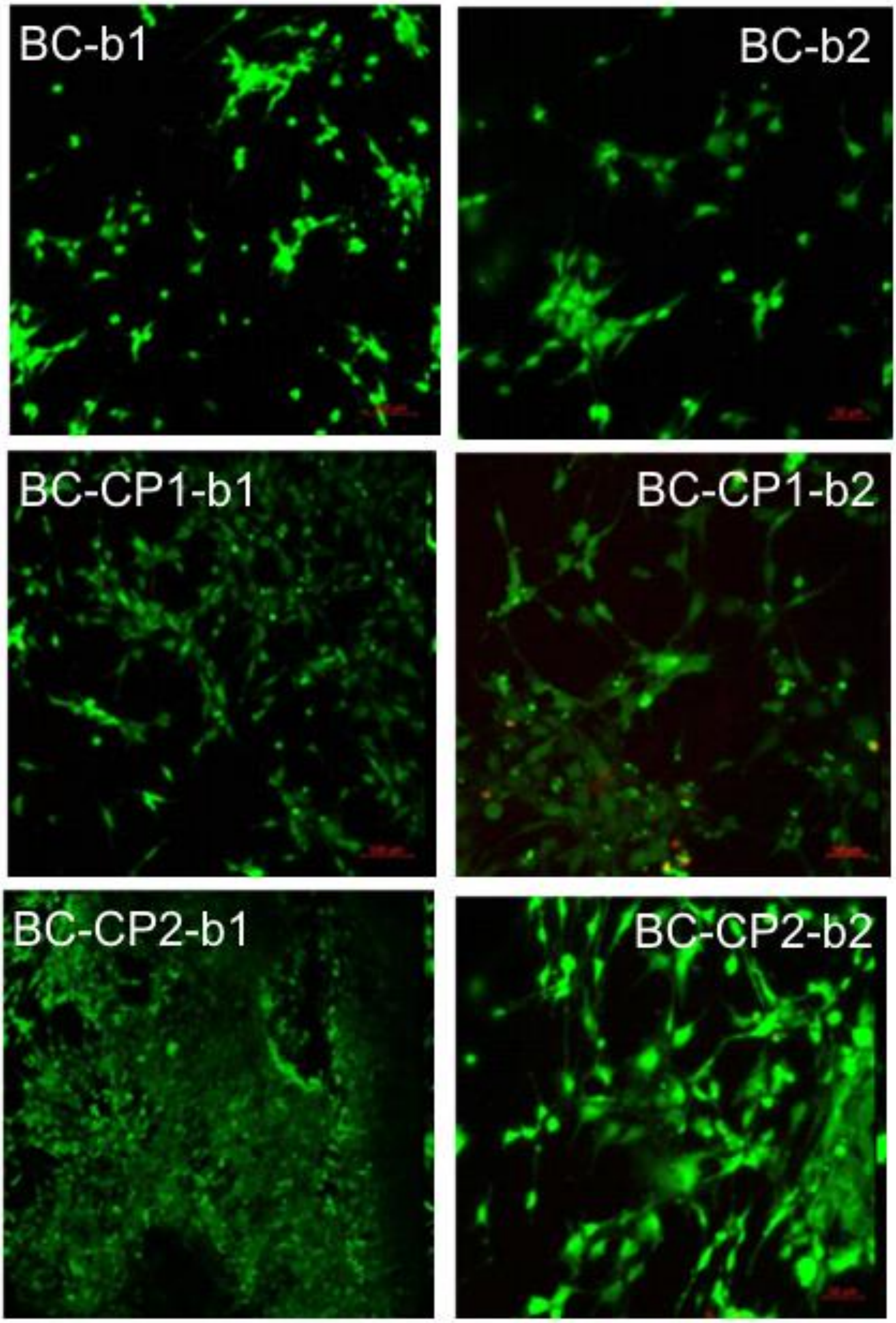

Figure S3. Fluorescence microscopy images at different magnifications: b1 (100μm) and b2 (50μm) of the adherent cells on BC, BC-CP1 and BC-CP2 samples after 7 days.

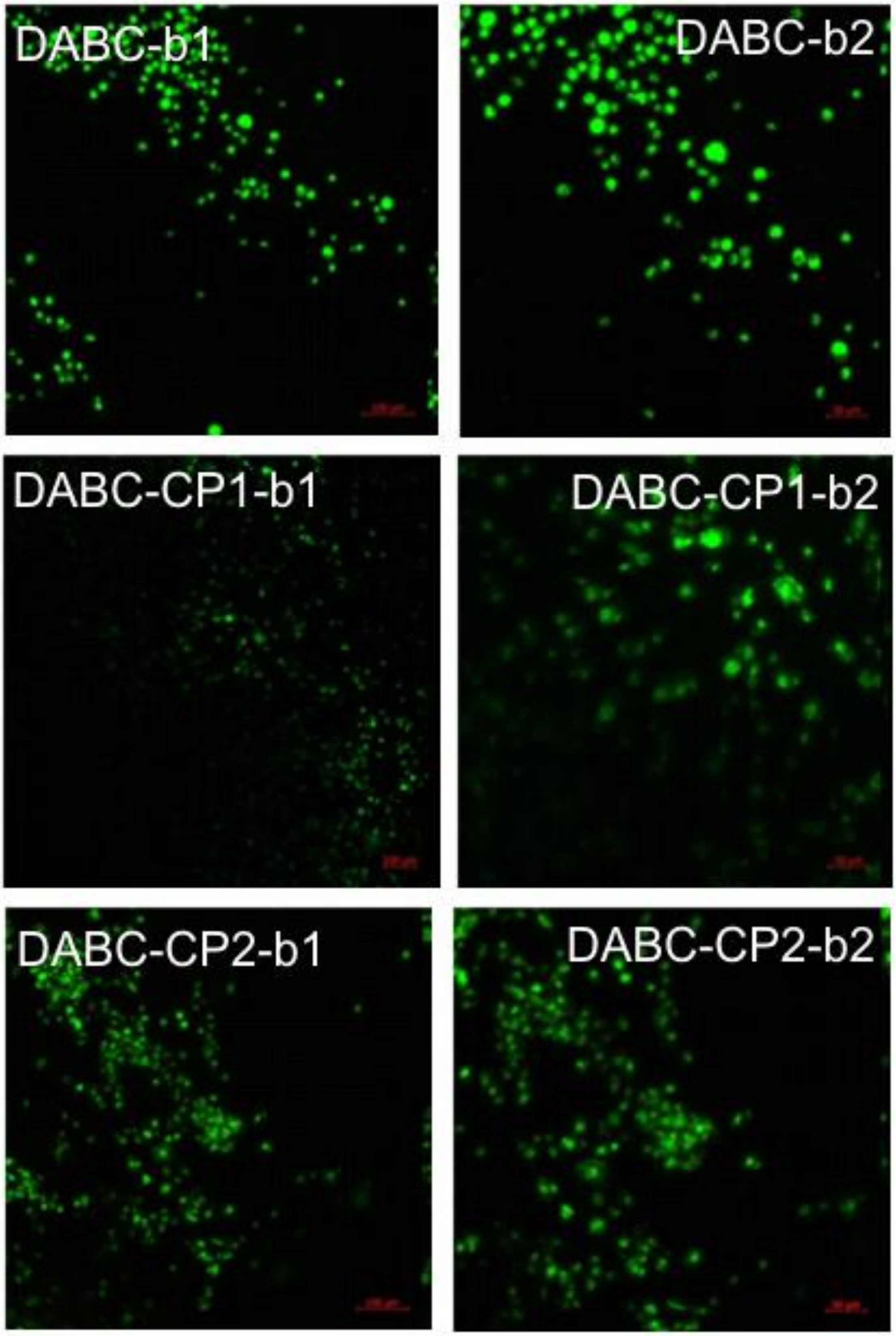

Figure S4. Fluorescence microscopy images at different magnifications: b1 (100μm) and b2 (50μm) of the adherent cells on DABC, DABC-CP1 and DABC-CP2 samples after 7 days.
